# Extracellular vesicles from glucocorticoids‐preconditioned synovial mesenchymal stem cells exert antiarthritic effects by downregulating the mRNA m6A modification of NLRP3 in macrophages through miR‐212‐5p

**DOI:** 10.1002/btm2.70042

**Published:** 2025-07-01

**Authors:** Xiaolong Shao, Ming Zhang, Shouye Hu, Zhi Yang

**Affiliations:** ^1^ Department of Joint Surgery, Xi'an Honghui Hospital Xi'an Jiaotong University Xi'an Shaanxi China; ^2^ Department of General Practice, Xi'an Honghui Hospital Xi'an Jiaotong University Xi'an Shaanxi China

**Keywords:** extracellular vesicles, glucocorticoids, N^6^‐methyladenosine, NLRP3, osteoarthritis, SMSCs

## Abstract

Osteoarthritis (OA) is a prevalent chronic degenerative joint disease with no known treatment for reversing its progression. However, recent studies have shown promising results for nano‐sized extracellular vesicles derived from preconditioned synovial mesenchymal stem cells (SMSCs) in treating various diseases, including OA. Glucocorticoids (GCs) possess potent anti‐inflammatory properties, but their long‐term use is limited due to potential adverse reactions. Building on previous research, this study aimed to investigate the therapeutic potential of extracellular vesicles secreted from GCs‐pretreated SMSCs (GCs‐EVs) in exerting antiarthritic effects. The results demonstrated that GCs‐EVs effectively inhibited cartilage degeneration and osteophyte formation in the OA mouse model by suppressing the release of inflammatory cytokines from synovial macrophages. This effect was attributed to the high expression of miR‐212‐5p in GCs‐EVs, which specifically inhibited the expression of methyltransferase‐like 3 (Mettl3). Consequently, the mRNA N6‐methyladenosine (m^6^A) level of nod‐like receptor pyrin domain 3 inflammasome (NLRP3) in macrophages was reduced, leading to decreased NLRP3 inflammasome activity and increased antiarthritic effects. Furthermore, in the co‐culture system, GCs‐EVs enhanced chondrocyte proliferation and migration while inhibiting chondrocyte apoptosis by suppressing the secretion of inflammatory factors by macrophages.

**Abbreviations**
ACAN: aggrecan
ADAMTS5: a disintegrin‐like and metalloproteinase domain with thrombospondin‐1 motifs 5
COL2A1: type II collagen alpha 1
DMM: destabilization of the medial meniscus
ECM: extracellular matrix
GCs: glucocorticoids
IL‐18: interleukin‐18
IL‐1*β*
: interleukin‐1β
Mettl3: methyltransferase‐like 3
MMP13: matrix metalloproteinase 13
MФ‐Mettl3^KD^
: knocking down Mettl3 in macrophages
Nig: nigericin
NLRP3: nod‐like receptor pyrin domain 3
OA: osteoarthritis
OARSI: osteoarthritis research society international
PMA: phorbol myristate acetate
TKA: total knee arthroplasty

**Translational Impact Statement**

Our research showed that GCs‐EVs significantly reduced cartilage degeneration and osteophyte formation in a mouse model of OA by decreasing the release of inflammatory cytokines from synovial macrophages. This effect is associated with increased levels of miR‐212‐5p in GCs‐EVs, which selectively downregulated Mettl3 expression. As a result, this downregulation led to a reduced m^6^A modification of NLRP3 in macrophages, lowering NLRP3 inflammasome activity and enhancing anti‐arthritic effects. This study highlights the superior anti‐arthritic properties of GCs‐EVs compared to conventional EVs and suggests a promising therapeutic approach for managing OA.

## 1 INTRODUCTION

Osteoarthritis (OA) is a common joint condition primarily affecting older adults, characterized by joint pain and stiffness. It arises from the degeneration of cartilage, the cushioning tissue between joints, and involves inflammation of the joint lining and underlying bone.^1^, ^2^ A key feature of OA is the dysregulation of cartilage cell homeostasis, accelerating disease progression.^3^ Macrophages, immune cells residing in the joint lining, play a pivotal role in maintaining immune homeostasis. Research has implicated inflammation as a major contributor to OA, driven by inflammatory molecules such as interleukin‐1*β* (IL‐1*β*) and interleukin‐18 (IL‐18). These cytokines promote cartilage degradation by stimulating the production of cartilage‐degrading enzymes.^4^, ^5^ The activation of the NOD‐like receptor family, pyrin domain containing 3 (NLRP3) inflammasome is a primary trigger for IL‐1*β* and IL‐18 production by macrophages.^6^, ^7^ This process is central to OA‐related inflammation, underscoring the importance of targeting these pathways for disease management.^8^, ^9^ Current OA treatments exhibit limited ability to halt disease progression.^10^, ^11^ Therefore, investigating novel approaches for OA prevention and management is imperative.

Recent research has indicated that mesenchymal stem cells (MSCs) hold significant promise for the treatment of various degenerative conditions,^12^, ^13^ including those affecting cartilage.^14^ Numerous animal studies and early‐stage human trials have demonstrated the efficacy of MSCs in promoting cartilage repair.^15^, ^16^ Among the various types of MSCs, synovial mesenchymal stem cells (SMSCs), derived from the synovial membrane, have gained significant attention since their discovery in 2001.^17^, ^18^ Their origin makes them particularly well‐suited for studies focused on cartilage repair. SMSCs are also advantageous due to their ease of extraction, rapid growth rate, and longevity, setting them apart from other stem cell types.^19^ However, the direct clinical application of stem cells is hindered by challenges such as concerns regarding genetic stability and potential immune system rejection.^20^, ^21^

Recent investigations have unveiled that the therapeutic efficacy of MSC therapy in cartilage repair is primarily attributed to the action of extracellular vesicles (EVs).^22^, ^23^, ^24^ These nanosized particles serve as vehicles for delivering specific bioactive molecules directly to target cells, facilitating tissue repair. Notably, EVs exhibit a favorable safety profile with minimal reported side effects, such as immune reactions or cancer risk.^25^, ^26^ Encapsulated within EVs are a diverse array of microRNAs (miRNAs), which play a critical role in modulating OA pathogenesis.^19^, ^27^, ^28^, ^29^ Preclinical and clinical studies have demonstrated that SMSC‐derived EVs play a pivotal role in OA treatment by stimulating cartilage cell regeneration, inhibiting cell apoptosis, and maintaining the extracellular matrix (ECM) homeostasis.^19^, ^28^, ^30^ These findings underscore the significant potential of EVs as a promising therapeutic strategy for OA.

Preconditioning techniques, such as exposure to cytokines, pharmacological agents, hypoxic environments, or physical stimuli, have been shown to enhance the efficacy of MSC transplantation by augmenting their biological functions and paracrine mechanisms.^31^ Glucocorticoids (GCs), renowned for their potent anti‐inflammatory properties, are commonly utilized to alleviate inflammation in individuals with OA.^32^, ^33^ However, the pleiotropic effects of GCs can result in various adverse effects, particularly when administered at higher doses or for prolonged durations. These side effects may include diabetes mellitus or glucose intolerance, hypertension, obesity, osteoporosis, and increased susceptibility to infections.^34^, ^35^, ^36^, ^37^, ^38^, ^39^ Therefore, we propose that preconditioning SMSCs with GCs may potentiate the antiarthritic benefits of their derived EVs (GCs‐EVs) while mitigating the negative consequences typically associated with GC therapy.

In the present study, we observed that GCs‐EVs exhibited superior anti‐arthritic properties compared to EVs derived from untreated SMSCs (EVs). This enhanced efficacy was primarily attributed to the elevated expression of microRNA‐212‐5p (miR‐212‐5p) within GCs‐EVs. MiR‐212‐5p could interact with methyltransferase‐like 3 (Mettl3), an RNA methyltransferase, leading to a reduction in Mettl3 expression. This decrease in Mettl3 resulted in lower levels of N6‐methyladenosine (m^6^A) modification on NLRP3 mRNA, ultimately leading to a decrease in the production of inflammatory cytokines and contributing to the observed anti‐arthritic effects. Our findings elucidate the underlying mechanisms by which GCs‐EVs exert their anti‐arthritic effects, thereby suggesting a promising therapeutic approach for the treatment of OA.

## 2 MATERIALS AND METHODS

### 2.1 Isolation and incubation of human chondrocytes

In this research, human chondrocytes were sourced from male patients who underwent total knee arthroplasty (TKA), following the acquisition of informed consent and approval from the Ethics Committee of Xi'an Honghui Hospital. During the TKA procedure, articular cartilage samples were collected and promptly placed in phosphate‐buffered saline (PBS) enriched with antibiotics. The cartilage tissue was then transported to the laboratory at a temperature of 4°C. Upon arrival, the tissue was diced into approximately 1 mm cubes and rinsed three times with antibiotic‐containing PBS. Following this, the cartilage cubes underwent digestion in PBS with 2% trypsin (Gibco) for 30 min, after which the trypsin solution was discarded. The cartilage underwent additional digestion with a complete culture medium containing 2 mg/mL type II collagenase (Gibco) for 12–16 h to isolate human chondrocytes. The harvested chondrocytes were then cultured in Dulbecco's Modified Eagle Medium/Nutrient Mixture F‐12 (DMEM/F12, Gibco), enriched with 20% fetal bovine serum (FBS) and 1% penicillin–streptomycin (Gibco).

### 2.2 Isolation and incubation of human SMSCs

In this research, human SMSCs were sourced from male patients who underwent TKA, following informed consent and approval from the Ethics Committee at Xi'an Honghui Hospital. During the TKA procedure, synovial tissue samples were collected and promptly placed in PBS with antibiotics. The samples were then transported to the laboratory at a temperature of 4°C. Upon arrival, the synovial tissue was rinsed three times with PBS to eliminate any debris and contaminants. Subsequently, the tissue was cut into small fragments using sterile medical scissors. These fragments were then transferred to a complete culture medium containing 2 mg/mL of type II collagenase (Gibco) and incubated for 12–16 h to facilitate the digestion and isolation of human SMSCs. The isolated SMSCs were maintained in low‐glucose Dulbecco's Modified Eagle Medium (DMEM) (Gibco), enriched with 10% fetal FBS and 1%penicillin‐streptomycin (Gibco). The culture medium was replaced after 12 hours and subsequently refreshed every 3 days. Upon reaching approximately 80% confluency, the cells were passaged. When the SMSCs reached the fifth generation, they were cryopreserved in liquid nitrogen for later use.

### 2.3 Isolation and incubation of mouse BMDMs

Male C57BL/6 mice femurs and tibias were isolated and disinfected by immersion in 70% ethanol. The ends of the bones were excised, and both ends were thoroughly rinsed with cold PBS using a 25‐G needle. The cells were cultured in DMEM enriched with 10% FBS, 1% penicillin/streptomycin, and 10% (v/v) conditioned medium from L929 mouse fibroblasts for a duration of 6 days, with medium changes every 3 days. Subsequently, the cells were centrifuged at 1000 × *g* for 5 min, resuspended in RPMI supplemented with 10% (v/v) FBS, and incubated at 37°C. For the inflammasome activation assay, the cells were primed with 200 ng/mL LPS (Sigma) for 4 h and then stimulated with 10 μM nigericin (Sigma) for 1 h.

### 2.4 Preparation and identification of extracellular vesicles

After subculturing the isolated SMSCs to the fifth generation, they were maintained in a serum‐free medium. Different groups of SMSCs were treated with dexamethasone (DEX) (Sigma‐Aldrich) and a vehicle control for a duration of 24 h. Following this treatment, the medium was replaced with fresh serum‐free medium, and the culture supernatant was collected after an additional 24 h. To isolate extracellular vesicles from the collected supernatant, a series of centrifugation steps were performed. Initially, the supernatant was centrifuged at 10,000 × *g* for 1 h to eliminate cellular debris. The resulting supernatant then underwent ultracentrifugation at 140,000 × *g* for 2 h, a process that was repeated twice to ensure thorough isolation of the extracellular vesicles. The size distribution of the isolated extracellular vesicles was analyzed using the NanoSight LM10 system (NanoSight Ltd., Novato, CA). The morphology of extracellular vesicles was further evaluated using a transmission electron microscope (TEM; Tecnai 12; Philips, Best, The Netherlands). To identify surface markers of these vesicles, a Western blot assay was conducted, targeting proteins such as CD9, CD63, Alix, Calnexin, and TSG101.

### 2.5 Experimental animals and procedures

In this research, male C57BL/6 mice, aged 8 weeks, were sourced from the Animal Medicine Center at West China Medical College, Sichuan University. Nlrp3^−/−^ mice were generously provided by Dr. Vishva M. Dixit from Genentech. All animal experiments were carried out in strict compliance with the guidelines established by the Animal Care and Use Committee of West China Hospital, and the study received approval from the Animal Protection and Ethics Committee of Sichuan University (Approval No. 20240303001). All animal studies meet ARRIVE guidelines. The mice were categorized into two groups—the operation group and the sham group—according to the requirements of the experiment. OA was induced via destabilization of the DMM in the right knee joint. In the operation group, both the anterior cruciate ligament and medial meniscus were excised during the DMM procedure, leading to decreased stability of the knee joint. Conversely, the sham group underwent a surgical incision in the skin and muscle without any removal of ligaments or menisci. To manage postoperative pain, all mice received buprenorphine at a dosage of 0.05 mg/kg. Additionally, 5 mg/kg of Gentamicin was administered to avert postoperative infections. Beginning 3 days post‐surgery, all mice engaged in forced jogging for 40 min daily on a specialized treadmill designed for mice, in accordance with their assigned groups. Throughout the OA model induction, miR‐212‐5p^KD^‐GCs‐EVs, GCs‐EVs, EVs, or PBS were injected into the knee joints of the mice on a weekly basis to assess their preventive effects against OA.

### 2.6 Intra‐articular injection of extracellular vesicles

Insulin injection syringes were utilized for drug administration. Mice undergoing destabilization of the DMM received injections of either PBS (10 μL), PBS‐EVs (10 μL, 10^11^ particles/mL), PBS‐GCs‐EVs (10 μL, 10^11^ particles/mL) or PBS‐miR‐212‐^KD^5p‐GCs‐EVs (10 μL, 10^11^ particles/mL) twice a week biweekly, based on their assigned group. Furthermore, throughout the 6‐week duration, all mice participated in jogging sessions lasting 40 min at a speed of 10 m/min on a designated treadmill.

### 2.7 Micro‐computed tomography (micro‐CT) joint imaging

Knee joint tissues from mice were examined using a Skyscan 1176 Micro‐CT system (Bruker, Billerica, MA, USA). 3D image reconstruction was performed with SkyScan volumetric NRecon software version 1.6 (Bruker, Billerica, MA, USA). Regions of interest (ROI) within the subchondral bone were identified following the 3D reconstruction. Osteophyte assessments were conducted by two blinded experimenters, unaware of the treatment groups.

### 2.8 Immunofluorescence staining

The tissues of the mice were perfused with 4% paraformaldehyde until they became firm. The knee joints were then dissected to eliminate the surrounding soft tissues. Following decalcification, dehydration, and wax embedding, the knee joint tissues were sliced into 5 μm thick sections for further experiments. After dewaxing and hydration, the tissue sections were incubated in 10% goat serum/PBST at room temperature for 1 h, then exposed to primary antibodies at 4°C overnight. After the primary antibody incubation, the sections underwent three washes with phosphate‐buffered saline with Tween (PBST), each lasting 10 min. Subsequently, the fluorescent secondary antibody was applied to the tissue sections and incubated at room temperature for 1 h. Following three additional washes with PBST, the sections were counterstained with DAPI to identify the cell nuclei. The sections were then mounted, sealed, and examined under a fluorescence microscope, with images captured for further analysis. The primary antibodies utilized in this study included anti‐COL2A1 (1:200, Abcam, ab34712), anti‐aggrecan (1:200, Proteintech, 13880‐1‐AP), anti‐ADAMTS5 (1:200; Abcam, ab182795), and anti‐MMP13 (1:200, Proteintech, 18165‐1‐AP).

### 2.9 Safranin‐O and fast green staining

The mouse knee tissue sections were dewaxed and hydrated in accordance with the manufacturer's guidelines for the Safranin‐O and Fast Green FCF Stain Kit (Solarbio). Following the staining process, these sections were examined under a microscope (Olympus, Japan) and documented through photography.

### 2.10 Enzyme‐linked immunosorbent assay (ELISA)

ELISA was employed to assess the concentrations of inflammatory markers in the knee tissues of mice, following established protocols. For the mouse knee tissue obtained, the excess femur and tibia were removed, and only the joint capsule and the joint head inside the joint capsule were retained. Immediately after weighing, liquid nitrogen was used for quick freezing. The RIPA buffer was added in a ratio of 1:10. The joint tissues were homogenized in RIPA buffer and subsequently centrifuged at 12,000 rpm for 30 min. The resulting supernatant was collected for ELISA analysis. Levels of IL‐1*β*, IL‐18, and TNF‐*α* were quantified using ELISA kits from Cloud‐Clone Corp (SEA563Hu, SEA064Hu, SEA133Hu).

### 2.11 Western blot analysis

Cells or extracellular vesicles were lysed with RIPA lysis buffer (Beyotime Biotechnology), and the mixture was centrifuged at 4°C for 15 min at 12,000 g to collect the supernatant. Subsequently, a 6× sodium dodecyl sulfate (SDS) loading buffer was incorporated, and the solution was heated at 100°C for 10 min. The proteins were then separated using SDS‐polyacrylamide gel electrophoresis and transferred to polyvinylidene fluoride membranes (Roche, Mannheim).

The sections were then treated with designated primary antibodies and suitable secondary antibodies according to the experimental protocols. After the antibody incubation, the sections were subjected to development and semi‐quantitative analysis. Primary antibodies used in this experiment included anti‐COL2A1 (1:2000, Abcam, ab34712), anti‐aggrecan (1:2000, Proteintech, 13,880‐1‐AP), anti‐ADAMTS5 (1:2000, Abcam, ab182795), anti‐MMP13 (1:2000, Proteintech, 18,165‐1‐AP), anti‐NLRP3 (1:1000, Adipogen, AG‐20B‐0014), anti‐IL‐1β (1:1000, R&D Systems, AF‐401‐NA), anti‐Ubiquitin (1:1000, Santa Cruz, sc‐8017), anti‐METTL3 (1:1000, Abcam, ab18810), anti‐Tubulin (1:1000, Proteintech, 11224‐1‐AP), anti‐Caspase‐1 (1:1000, Abcam, ab108362), anti‐CD9 (1:2000, Abcam, ab223052), anti‐CD63 (1:2000, Abcam, ab134045), anti‐CD81 (1:2000, Abcam, ab109201), anti‐Alix (1:1500, Proteintech, 12422‐1‐AP), anti‐tubulin (1:2000, Proteintech, 10094‐1‐AP), anti‐TSG101 (1:2000, Proteintech, 14497‐1‐AP), anti‐Calnexin (1:2000, Proteintech, 10427‐2‐AP) and anti‐Ago2 (1:2000, Abcam, ab186733).

### 2.12 Measurement of m^6^A modification

The levels of m^6^A in RNA were assessed using an established methodology. In summary, total RNA was extracted from cells and subjected to DNase treatment. The total m^6^A RNA was analyzed utilizing the m^6^A RNA methylation assay kit (Abcam, ab185912), following the manufacturer's instructions. For the assay, the wells were coated with 200 ng of RNA. A suitably diluted solution of capture and detection antibodies was added to the test wells. The m^6^A levels were quantified via a calorimetric approach, measuring absorbance at 450 nm, and were calculated using standard curves.

### 2.13 Transfection of small interfering RNA

Small interfering RNAs (siRNAs) designed to target METTL3 (siRNA‐METTL3) and Ago2 (siRNA‐Ago2), along with their corresponding scrambled control siRNAs (siRNA‐NC), were obtained from TSINGKE (Nanjing, China). Transfection of cells was performed using Lipofectamine 2000 reagent (Invitrogen), following the guidelines provided by the manufacturer.

### 2.14 MiRNA microarray assay

The microRNA microarray analysis for EVs and GCs‐EVs was conducted by OE Biotech Company in Shanghai, China. Each type of extracellular vesicle was analyzed using 3 samples. The fragmentation mixtures were processed using the Agilent Human microRNA Array 21.0. Furthermore, the microarray analysis was performed on the Affymetrix miRNA 4.0 platform located in Santa Clara, California, USA. Sample labeling, microarray hybridization, and washing procedures adhered to the manufacturer's guidelines provided by Agilent Technologies Inc. Differentially expressed microRNAs were determined using a fold change threshold of ≥1.5 for both upregulated and downregulated genes.

### 2.15 Luciferase reporter assay

Sequences corresponding to the 3′‐UTR of the METTL3 mRNA, featuring either the wild‐type (WT) or mutated (MUT) miR‐212‐5p binding sites, were synthesized by GeneScript (Nanjing, China). These sequences were subsequently cloned into the FseI and XbaI restriction sites of the pGL3 luciferase control reporter vector (Promega, USA), resulting in the creation of the METTL3 3′‐UTR reporter constructs, namely pGL3‐WT‐METTL3 and pGL3‐MUT‐METTL3. On the day designated for transfection, the engineered pGL3 luciferase control reporter vector was introduced into 293T cells in accordance with the experimental protocol. After 24 h post‐transfection, the expression of fluorescent marker genes within the cells was examined using fluorescence microscopy. The luciferase activities of both Firefly and Renilla were assessed utilizing the Dual‐Luciferase® Assay Kit (Promega, Madison, WI, USA).

### 2.16 Proliferation assay of chondrocytes

Initially, a co‐culture system was developed involving chondrocytes alongside either NC^KD^ macrophages or METTL3^KD^ macrophages. Depending on the experimental design, PBS, EVs, or GCs‐EVs were introduced. Following a 24‐h co‐culture period, the experiment proceeded in accordance with the guidelines of the BeyoClick™ EdU‐594 Cell Proliferation Detection Kit (Beyotime). Chondrocytes were first digested using trypsin to create a cell suspension. The proliferation rate of chondrocytes across all groups was assessed via flow cytometry, following the kit's instructions. Additionally, chondrocytes from each group were examined and photographed using fluorescence microscopy.

### 2.17 Apoptosis assay of chondrocytes

A co‐culture system involving chondrocytes and either NC^KD^ or METTL3^KD^ macrophages was established as previously described. Depending on the experimental framework, PBS, EVs, or GCs‐EVs were introduced. Following a 24‐h co‐culture period, chondrocytes were digested with trypsin to isolate suspension cells. The apoptosis rates of chondrocytes across all groups were assessed using flow cytometry, in accordance with the Annexin V‐FITC/PI Apoptosis Detection Kit (Vazyme) protocol. To enhance the objectivity and reliability of the results, chondrocytes from each group were also evaluated using the TUNEL Bright Green Apoptosis Detection Kit (Vazyme). This involved testing for TUNEL‐positive cells, which were subsequently observed and photographed under a fluorescence microscope.

### 2.18 Migration assay of chondrocytes

In the scratch assay, a co‐culture system was developed in accordance with the procedures outlined in the proliferation assay section. To eliminate the influence of cell proliferation on the results, cells were incubated for 48 h. Prior to the experiment, chondrocytes were treated with mitomycin at a concentration of 1 μg/mL for 1 h. After a 24‐h incubation period, a sterile 200 μL pipette tip was utilized to create a scratch in the cell layer. The cells were then washed three times with PBS, and images were captured at 0, 24, and 48 h using a microscope.

To improve the reliability of our experimental findings, we implemented a dual verification strategy. Macrophages were cultured in 24‐well plates to create a co‐culture system. Following a 24‐h incubation period, chondrocytes were fixed using 4% paraformaldehyde for 15 min, subsequently stained with 0.5% crystal violet for 30 min, and rinsed three times with PBS. The upper surface of the upper chamber was swabbed to eliminate any non‐migrated cells. The migration rates of chondrocytes in each group were examined under a microscope, with four random fields selected for further analysis.

### 2.19 Statistical analyses

All experiments were conducted with a minimum of three independent biological replicates. The data are presented as mean ± standard deviation. Statistical analyses were performed using GraphPad software version 7.0 and SPSS version 19.0. For comparisons between two groups, the Student's *t*‐test was employed, while one‐way or two‐way ANOVA was utilized for comparisons involving more than two groups to evaluate the significance of differences. A *p*‐value of less than 0.05 was considered statistically significant.

## 3 RESULTS

### 3.1 Identification of SMSCs

**FIGURE 1:**
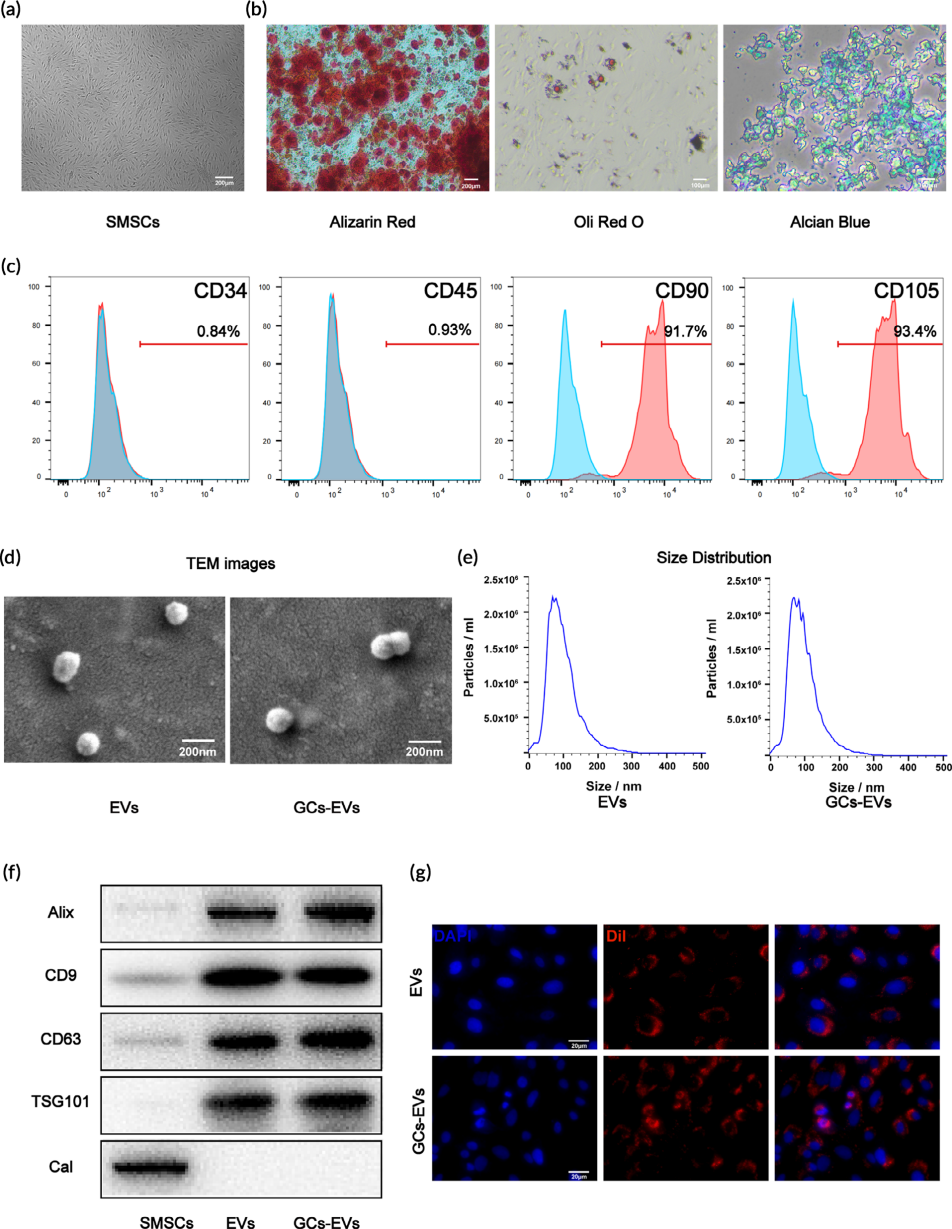
Identification of human SMSCs and extracellular vesicles. (a) SMSCs exhibited a representative spindle‐like morphology. Scale bar = 200 μm. (b) SMSCs show the multidirectional differentiation potential of osteogenesis, adipogenesis, and chondrogenesis. Scale bar = 200 μm for osteogenesis; Scale bar = 100 μm for adipogenesis and chondrogenesis. (c) The characteristic cell surface markers of SMSCs were analyzed by flow cytometry. (d) TEM images of EVs and GCs‐EVs. (e) The particle size distributions of EVs and GCs‐EVs were analyzed by NanoSight. (f) Extracellular vesicle markers (CD9, CD63, Alix, TSG101, Calnexin) analyzed by western blot. (g) Chondrocyte internalization of EVs and LPS‐pre‐EVs as observed by fluorescence microscopy. Scale bar = 20 μm.

In the “Methods” section, we detailed the isolation of SMSCs from knee joint tissue procured during TKA. The isolated SMSCs were cultured, and cells at the fifth passage were utilized for subsequent experiments. Microscopic examination revealed that the SMSCs exhibited a characteristic spindle‐shaped morphology (Figure 1a). To induce differentiation, the cultured SMSCs were exposed to osteogenic, adipogenic, and chondrogenic media. Differentiation was confirmed through Alizarin Red, Oil Red O, and Alcian Blue staining, which indicated osteogenic (Figure 1b, left panel), adipogenic (Figure 1b, middle panel), and chondrogenic (Figure 1b, right panel) differentiation, respectively. These results collectively demonstrated the multipotent differentiation potential of the isolated SMSCs, a hallmark characteristic of MSCs. Additionally, flow cytometry analysis was employed to assess the expression of surface marker proteins on the isolated SMSCs. The analysis revealed that nearly all cells were negative for CD34 and CD45 while positive for CD90 and CD105 surface markers (Figure 1c). These findings collectively confirm the identity of the isolated cells as SMSCs.

Next, EVs and GCs‐EVs were isolated from the supernatants of SMSCs and GCs‐SMSCs, respectively. Transmission electron microscopy revealed that both types of vesicles exhibited a round or disk‐like morphology (Figure 1d), consistent with the typical appearance of extracellular vesicles. Additionally, NanoSight analysis indicated that the diameters of the EVs and GCs‐EVs predominantly ranged from 50 to 200 nm(Figure 1e), aligning with the established size distribution of extracellular vesicles. Western blot analysis confirmed the presence of extracellular vesicle markers, including CD9, CD63, Alix, and TSG101, in both EVs and GCs‐EVs, while these markers were absent in SMSCs (Figure 1f). Furthermore, the negative extracellular vesicle marker calnexin was not detected. To visualize the uptake of EVs and GCs‐EVs by chondrocytes, we pre‐labeled the vesicles with Dil dye and co‐cultured them with chondrocytes. Fluorescence microscopy demonstrated that the chondrocytes could internalize these extracellular vesicles (Figure 1g).

### 3.2 In the DMM‐induced OA mouse model, GCs‐EVs demonstrated superior efficacy in inhibiting OA progression compared to EVs

**FIGURE 2:**
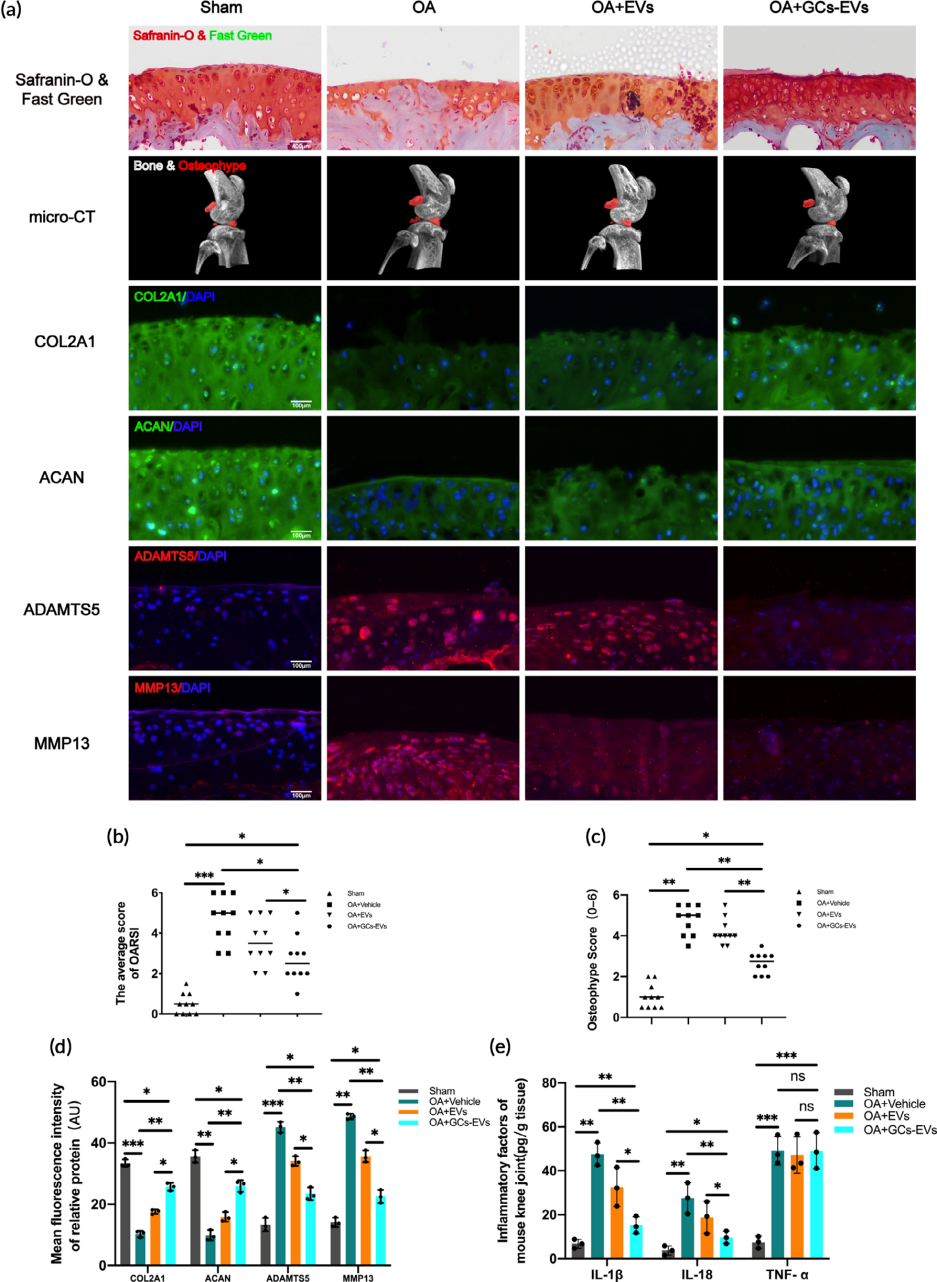
GCs‐EVs has a better effect on preventing the disease progression of knee osteoarthritis. (a) Severity of DMM‐induced OA mice model as determined by Safranin‐O andFast Green, and micro‐CT analysis. Expression levels of COL2A1, aggrecan, MMP13 and ADAMTS5 as determined by immunofluorescence staining. Scale bar = 400 μm for Safranin‐O and Fast Green staining; Scale bar = 100 μm for immunofluorescence staining. (b) OARIS scores in each group (*n* = 10, one‐way ANOVA). (c) Osteophyte score of each group (*n* = 10, one‐way ANOVA). (d) Quantification of mean fluorescence intensity of COL2A1, ACAN, ADAMTS5 and MMP13 in each group (*n* = 3, one‐way ANOVA). (e) Levels of IL‐1*β*, IL‐18 and TNF‐*α* in mice knee tissue as determined by ELISA (*n* = 3, one‐way ANOVA). Data are presented as mean ± SD. ns = no significance, *p < 0.05, **p < 0.01, ***p < 0.001.

Following the establishment of a DMM‐induced OA mouse model to investigate the potential inhibitory effects of GCs‐EVs on OA progression, knee sections from each experimental group underwent Safranin‐O and Fast Green staining. This staining technique enabled the visualization of structural alterations in the articular cartilage, subchondral bone, and surrounding bone tissue. The results revealed that the cartilage layer in the GCs‐EVs group was significantly thicker and smoother than that in the EVs group, as depicted in Figure 2a. Additionally, the severity of OA, as assessed using the Osteoarthritis Research Society International (OARSI) scoring system, was significantly reduced in the GCs‐EVs group relative to the EVs group (Figure 2b). Furthermore, micro‐CT imaging was employed to evaluate osteophyte formation in the mice across all experimental groups. The micro‐CT analysis revealed that the DMM‐induced OA group exhibited a significantly greater degree of osteophyte formation than the sham group, thus confirming the successful induction of the OA model. Notably, the osteophytes observed in the GCs‐EVs group were significantly smaller than those in the EVs group (Figure 2a). Furthermore, the osteophyte score for the GCs‐EVs group was considerably lower than that for the EVs group (Figure 2c), suggesting that GCs‐EVs are more effective in inhibiting osteophyte development in the DMM‐induced OA mouse model than EVs.

It is well‐established that ECM degradation is a critical pathological hallmark of OA.^40^ To investigate the impact of GCs‐EVs on ECM proteins (COL2A1 and ACAN) and ECM‐degradation‐associated proteases (MMP13 and ADAMTS5), immunofluorescence analysis was performed on knee sections from mice across all experimental groups. The results demonstrated a significant decrease in ECM protein levels in the OA group compared to the sham group, accompanied by a significant increase in ECM‐degradation‐related proteases, confirming successful model establishment. Furthermore, mice treated with GCs‐EVs exhibited a significant increase in ECM protein levels compared to those in the EVs group, while the expression of ECM‐degradation‐related proteases was significantly decreased (Figure 2a, d). These findings suggest that GCs‐EVs effectively inhibit the expression of proteases involved in ECM degradation, thereby protecting ECM proteins from deterioration.

Current research indicates that OA is characterized by a distinct inflammatory response.^41^ As outlined in the Methods section, ELISA was performed to quantify the levels of inflammatory factors in the knee tissues of mice across various groups. The results demonstrated a significant upregulation of IL‐1*β*, IL‐18, and TNF‐*α* following DMM surgery. Importantly, treatment with EVs and GCs‐EVs resulted in a downregulation of IL‐1*β* and IL‐18, while TNF‐*α* levels remained relatively unchanged (Figure 2e). Furthermore, the GCs‐EVs group exhibited lower levels of IL‐1*β* and IL‐18 compared to the EVs group (Figure 2e). These findings suggest that GCs‐EVs are more potent in suppressing the expression of inflammatory cytokines.

### 3.3 The progression of OA was inhibited by GCs‐EVs in the DMM‐induced OA NLRP3 knockout mice model

**FIGURE 3:**
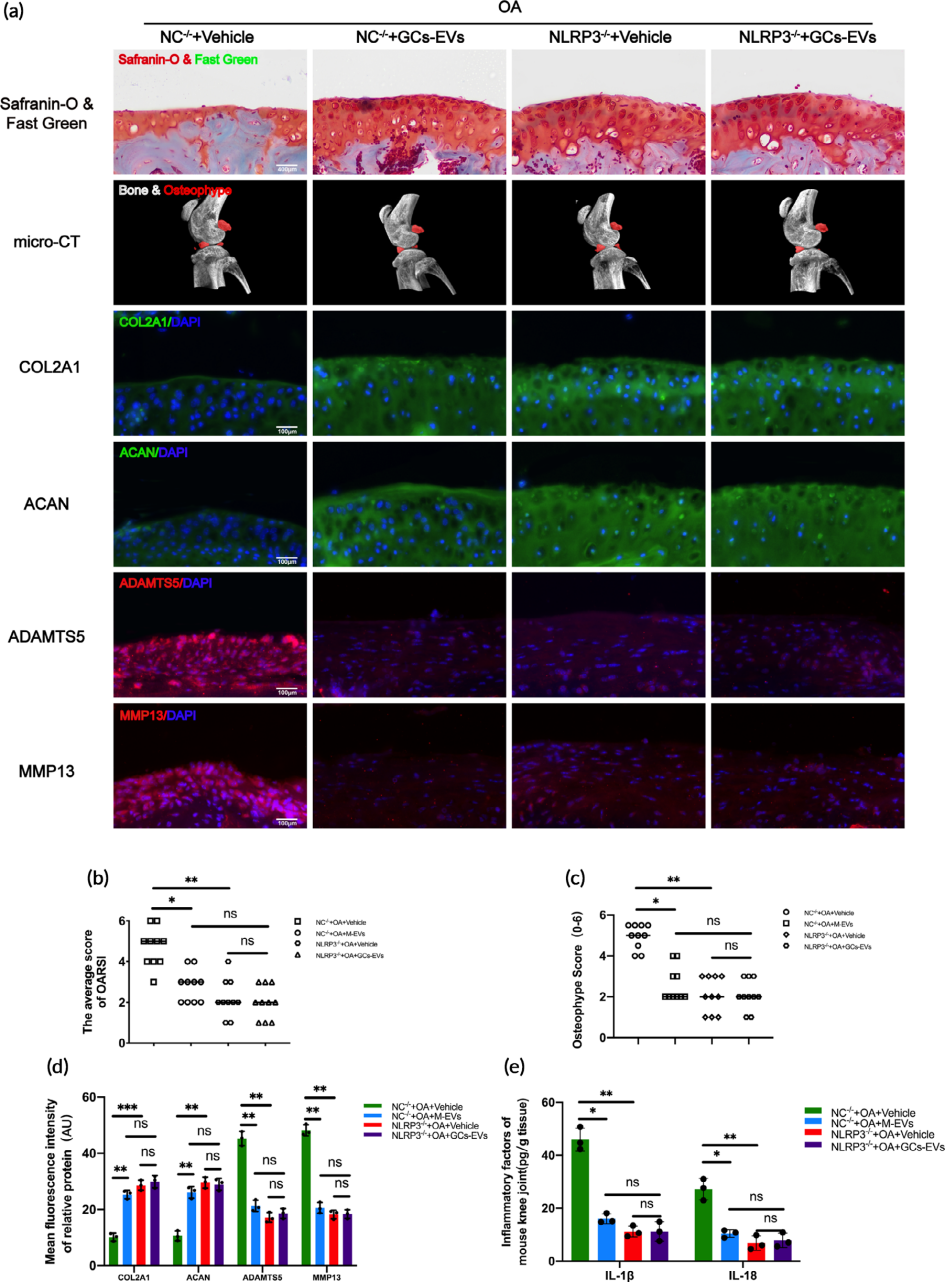
In NLRP3^−/−^ mice, the role of GCs‐EVs in preventing the progression of OA disappears. (a) Severity of DMM‐induced OA mice model as determined by Safranin‐O and Fast Green, and micro‐CT analysis. Expression levels of COL2A1, ACAN, MMP13, and ADAMTS5 as determined by immunofluorescence staining. Scale bar = 400 μm for Safranin‐O and Fast Green staining; Scale bar = 100 μm for immunofluorescence staining. (b) OARIS scores in each group (*n* = 10, one‐way ANOVA). (c) Osteophyte score of each group (*n* = 10, one‐way ANOVA). (d) Quantification of mean fluorescence intensity of COL2A1, aggrecan, ADAMTS5, and MMP13 in each group (*n* = 3, one‐way ANOVA). (e) Levels of IL‐1*β* and IL‐18 in mice knee tissue as determined by ELISA (*n* = 3, one‐way ANOVA). Data are presented as mean ± SD. ns = no significance, *p < 0.05, **p < 0.01.

As IL‐1*β* and IL‐18 are key inflammatory mediators activated by the NLRP3 inflammasome, they may serve as indicators of NLRP3 inflammasome activity to some extent.^42^ Consequently, we hypothesized that GCs‐EVs could exert their anti‐arthritic effects by modulating the activity of the NLRP3 inflammasome. To evaluate this hypothesis, we induced an OA model in NLRP3 knockout mice (NLRP3^−/−^) via DMM surgery. Post‐surgery observations revealed that the cartilage layer's thickness and smoothness in NLRP3^−/−^ mice remained largely unchanged, irrespective of GCs‐EVs treatment (Figure 3a). Similarly, the OARSI scores did not show significant variation (Figure 3b). Micro‐CT analyses further indicated that the absence of NLRP3 in mice did not significantly influence the size of osteophyte formation or the osteophyte scores across all treatment groups (Figure 3a, c). Immunofluorescence assay results indicated that the knockout of NLRP3 in mice abolished the inhibitory effects of GCs‐EVs on the expression of ECM‐degradation‐related proteases, which are crucial for protecting ECM proteins from degradation (Figure 3a, d). As expected, the levels of IL‐1*β* and IL‐18 in the knee joints of NLRP3^−/−^ mice, regardless of GCs‐EVs treatment, showed no significant variation, mirroring the inflammatory cytokine levels observed in NC^−/−^ mice treated with GCs‐EVs (Figure 3e). Overall, these findings support our hypothesis that GCs‐EVs exert an antiarthritic effect through the modulation of NLRP3 inflammasome activity.

### 3.4 GCs‐EVs reduce the mRNA m^6^A level of NLRP3 in macrophages

**FIGURE 4:**
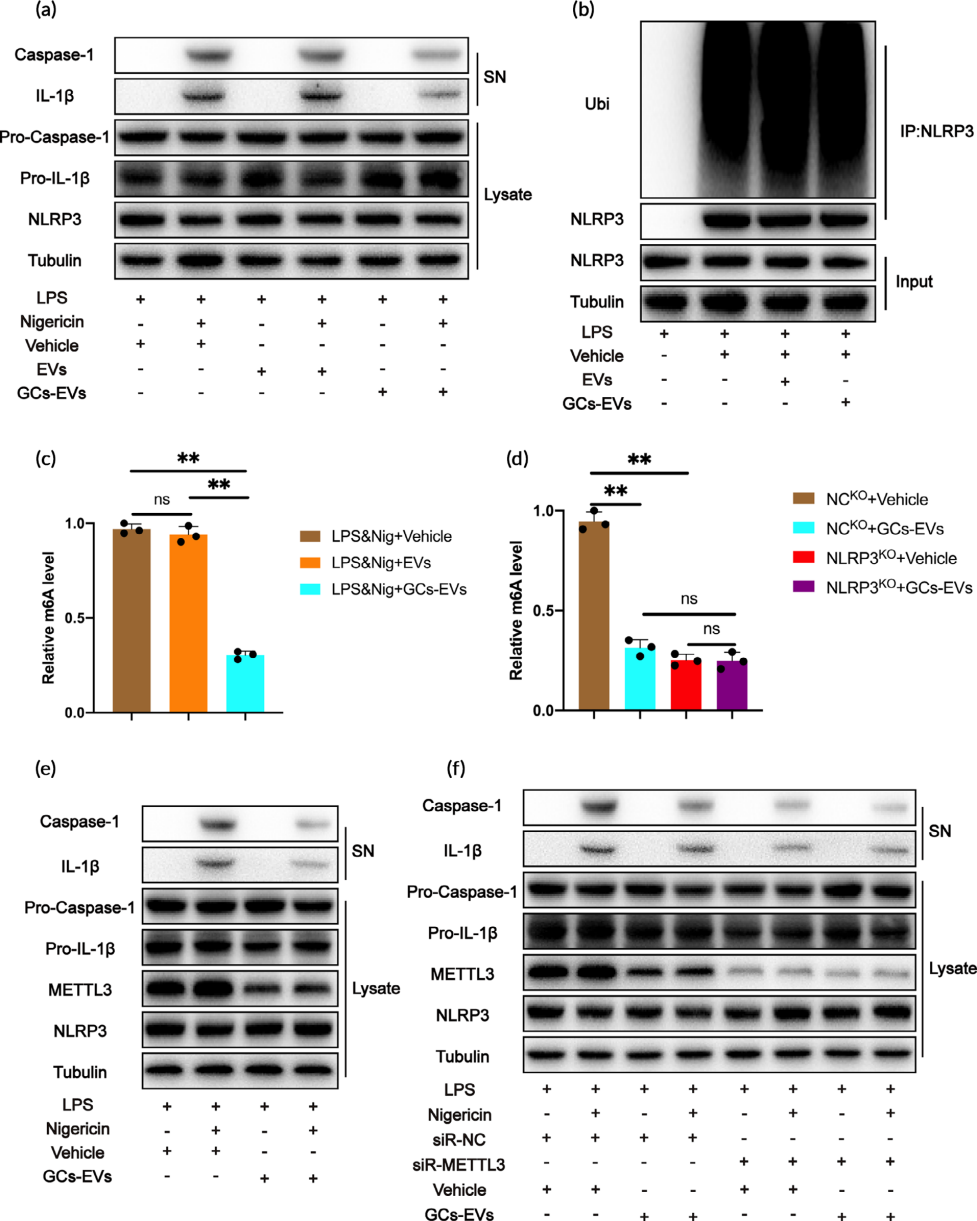
GCs‐EVs reduce the mRNA m^6^A level of NLRP3 by METTL3. (a) Western blotting analysis of IL‐1*β* and cleaved caspase‐1 levels in culture SN and pro‐IL‐1*β*, pro‐caspase‐1, and NLRP3 levels in mice macrophage lysates in each group. (b) NLRP3 ubiquitination was analyzed in mice macrophages with different treatments. (c) The mRNA m^6^A level of NLRP3 in each group. (d) The mRNA m^6^A level of NLRP3 in each group. (e) Western blotting analysis of IL‐1*β* and cleaved caspase‐1 levels in culture SN and pro‐IL‐1*β*, pro‐caspase‐1, NLRP3, and METTL3 levels in mice macrophage lysates in each group. (f) Western blotting analysis of IL‐1*β* and cleaved caspase‐1 levels in culture SN and pro‐IL‐1*β*, pro‐caspase‐1, NLRP3, and METTL3 levels in NC^KD^ mice macrophage lysates and METTL3^KD^ mice macrophage lysates in each group. ns = no significance, **p < 0.01.

Following our in vivo investigations demonstrating the anti‐arthritic effects of GCs‐EVs through modulation of the NLRP3 inflammasome, we sought to elucidate the underlying mechanisms by which GCs‐EVs influence NLRP3 activity. Initially, primary macrophages were isolated from mice, activated to induce NLRP3 inflammasome activation, and treated with either EVs or GCs‐EVs. The levels of downstream proteins IL‐1*β* and Caspase‐1, markers of NLRP3 inflammasome activity, were then measured. The results indicated that GCs‐EV treatment led to a reduction in the expression levels of both IL‐1*β* and Caspase‐1, consistent with our in vivo findings (Figure 4a). Given the well‐established correlation between NLRP3 inflammasome activity and its ubiquitination levels,^43^, ^44^, ^45^ we hypothesized that GCs‐EVs might modulate NLRP3 inflammasome activity through the regulation of its ubiquitination. To validate this hypothesis, we assessed the ubiquitination levels of the NLRP3 inflammasome in primary macrophages treated with either EVs or GCs‐EVs. However, no significant differences in NLRP3 ubiquitination levels were observed between the two groups (Figure 4b). Recent studies have suggested that modulation of NLRP3 mRNA m^6^A methylation can influence its activity.^46^ Therefore, we proposed that GCs‐EVs might modulate NLRP3 inflammasome activity through the regulation of its mRNA m^6^A levels. To investigate this further, we employed an m^6^A RNA methylation assay kit to quantify the m^6^A levels of NLRP3 mRNA in different treatment groups. The results demonstrated that GCs‐EVs treatment led to a reduction in the m^6^A levels of NLRP3 mRNA in primary macrophages (Figure 4c). Additionally, we isolated primary macrophages from NLRP3^−/−^ mice and observed no significant differences in NLRP3 m^6^A levels between macrophages treated with or without GCs‐EVs post‐NLRP3 knockout (Figure 4d). These findings strongly support our in vivo observations.

### 3.5 GCs‐EVs reduce the mRNA m6A levels of NLRP3 in macrophages by suppressing Mettl3 expression

Numerous prior investigations have indicated a strong correlation between the m^6^A modification level of NLRP3 mRNA and the expression levels of Mettl3.^46^, ^47^, ^48^, ^49^ Based on this evidence, we hypothesized that GCs‐EVs might influence the m^6^A modification of NLRP3 mRNA by modulating Mettl3 expression. To validate this hypothesis, we analyzed Mettl3 expression in primary macrophages derived from mice. Our findings revealed that treatment with GCs‐EVs led to a reduction in the levels of inflammatory proteins IL‐1*β* and Caspase‐1, downstream targets of NLRP3, as well as a decrease in Mettl3 expression (Figure 4e). Furthermore, we employed siRNA to silence Mettl3 in macrophages. Subsequent treatment, regardless of the presence of GCs‐EVs, did not significantly alter the expression levels of the downstream inflammatory proteins IL‐1*β* and Caspase‐1 associated with NLRP3 (Figure 4f). These results reinforce our hypothesis, suggesting that GCs‐EVs can decrease the methylation levels of NLRP3 by inhibiting Mettl3 expression. This, in turn, leads to a reduction in NLRP3 activity and contributes to their anti‐inflammatory effects.

### 3.6 GCs‐EVs decrease the mRNA m^6^A modification of NLRP3 by suppressing Mettl3 expression via the upregulation of miR‐122‐5p

**FIGURE 5:**
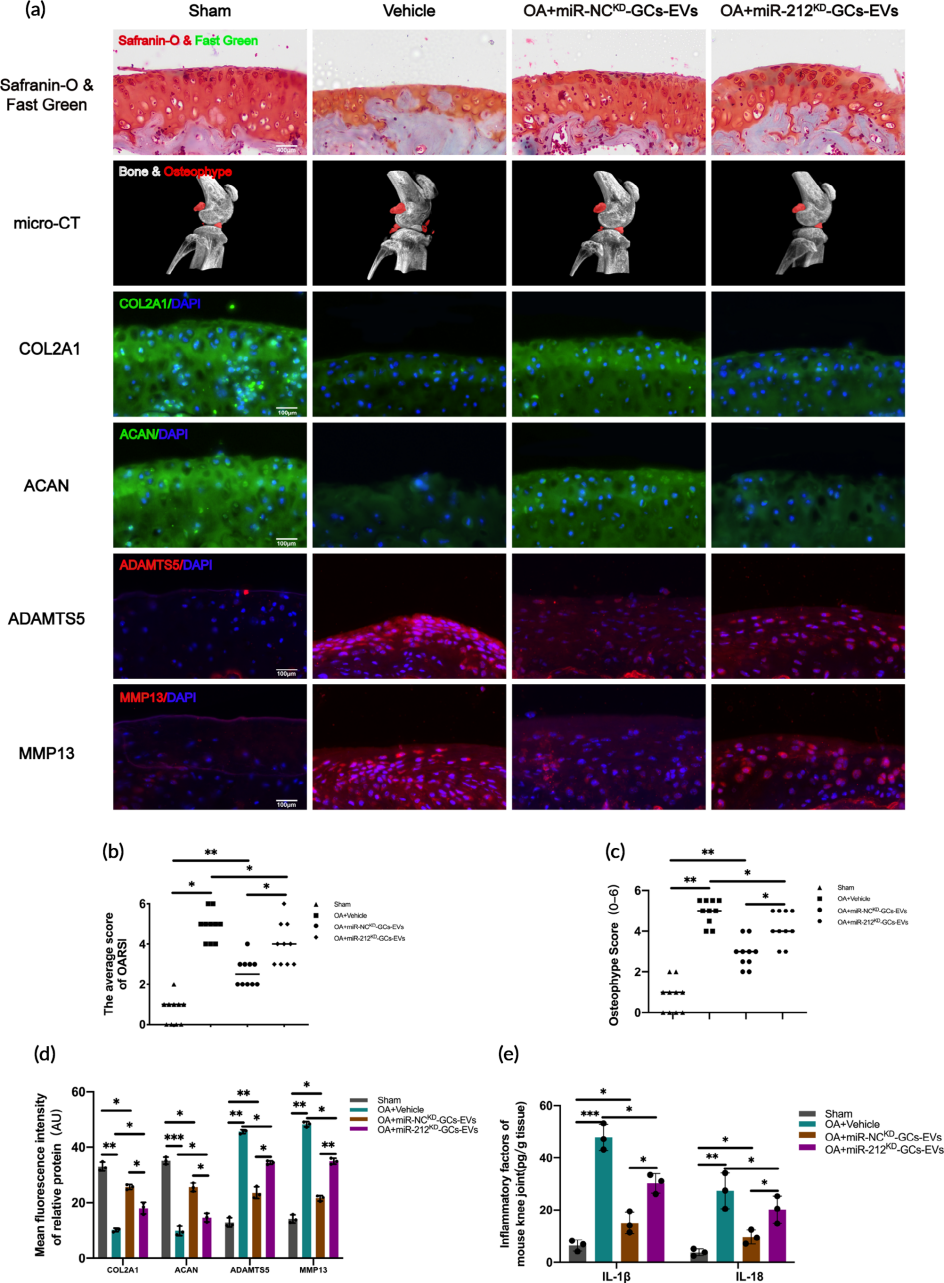
GCs‐EVs exert anti‐arthritic effects by targeted inhibiting Mettl3 expression with high expression of miR‐212‐5p. (a) Ago2 protein expression in SMSCs was detected by western blot. (b)The mRNA m^6^A level of NLRP3 in each group. (c) Western blotting analysis of IL‐1*β* and cleaved caspase‐1 levels in culture SN and pro‐IL‐1*β*, pro‐caspase‐1, NLRP3, and METTL3 levels in mice macrophage lysates treated with either NC^KD^‐GCs‐EVs or Ago2^KD^‐GCs‐EVs. (d) Heat map of miRNA levels between EVs and GCs‐EVs groups (*n* = 3, Student's *t*‐test). (e) Comparisons of the five most significantly upregulated miRNAs between EVs and GCs‐EVs. (f) The target sequence of miR‐212‐5p estimated within 3′‐UTR of Mettl3. (g) Inhibitor‐miR‐212‐5p was transfected into SMSCs, and RT‐PCR was performed to evaluate transfection efficiency (*n* = 6, Student's *t‐*test). (h) RT‐PCR was used to detect the expression level of miR‐212‐5p in GCs‐EVs (*n* = 6, Student's *t*‐test). (i) Western blotting analysis of IL‐1*β* and cleaved caspase‐1 levels in culture SN and pro‐IL‐1*β*, pro‐caspase‐1, NLRP3, and METTL3 levels in mice macrophage lysates treated with either NC^KD^‐GCs‐EVs or miR‐212‐5p^KD^‐GCs‐EVs. (j) Western blotting analysis of IL‐1*β* and cleaved caspase‐1 levels in culture SN and pro‐IL‐1*β*, pro‐caspase‐1, NLRP3, and METTL3 levels in THP‐1 cell lysates treated with either NC^KD^‐GCs‐EVs or miR‐212‐5p^KD^‐GCs‐EVs. (k) Luciferase reporter assay showing that METTL3 is the target gene for miR‐212‐5p (*n* = 3, Student's *t*‐test). Data are presented as mean ± SD. ns = no significance, **p < 0.01, ***p < 0.001.

Extracellular vesicles are known to encapsulate a diverse array of nucleic acids, including prominent RNA types such as mRNA, miRNA, and ribosomal RNA (rRNA), alongside various non‐coding RNAs like tRNA fragments, piwi‐interacting RNA, vault RNA, and Y RNA.^50^, ^51^, ^52^, ^53^, ^54^, ^55^ In the context of OA treatment, numerous studies have demonstrated that miRNAs within extracellular vesicles contribute to therapeutic effects through multiple pathways.^27^, ^56^, ^57^ Consequently, we hypothesized that GCs‐EVs exert their therapeutic effects through the miRNAs they carry. To validate this hypothesis, we employed siRNA to downregulate Argonaut‐2 (Ago‐2) protein in SMSCs (Figure 5a). Ago2 is a pivotal regulator of miRNAs, influencing miRNA‐mediated mRNA cleavage and translational inhibition.^58^ Ago2^KD^‐GCs‐EVs derived from Ago2^KD^‐SMSCs were isolated, and macrophages were subsequently exposed to either NC^KD^‐GCs‐EVs or Ago2^KD^‐GCs‐EVs. The results indicated that the knockdown of *Ago2* abolished the initial suppressive impact of GCs‐EVs on the mRNA m^6^A levels of NLRP3 and its downstream inflammatory mediators, IL‐1*β* and Caspase‐1 (Figure 5b, c). This finding suggests that the anti‐arthritic properties of GCs‐EVs are mediated through their miRNA cargo.

Based on these findings, we conducted a comprehensive analysis of miRNA expression profiles in EVs and GCs‐EVs, identifying a subset of differentially expressed miRNAs (Figure 5d). Among these, five miRNAs—miR‐36, miR‐212‐5p, miR‐4467, miR‐5, and miR‐8—exhibited the most significant expression variations (Figure 5e). Further bioinformatic analysis using TargetScan revealed that only miR‐212‐5p had the potential to target the mRNA of *Mettl3*. Notably, miR‐212‐5p was significantly upregulated in GCs‐EVs. We also predicted the specific interaction sequence between miR‐212‐5p and Mettl3 mRNA (Figure 5f). Consequently, we hypothesized that the elevated levels of miR‐212‐5p in GCs‐EVs could suppress *Mettl3* expression, contributing to their anti‐arthritic properties. To validate this hypothesis, we employed a specific miR‐212‐5p inhibitor to knock down miR‐212‐5p expression in SMSCs, subsequently collecting the resulting miR‐212‐5p^KD^‐GCs‐EVs. The expression levels of miR‐212‐5p in miR‐212‐5p^KD^‐SMSCs and miR‐212‐5p^KD^‐GCs‐EVs were assessed using RT‐PCR (Figure 5g, h). Our results demonstrated that the knockdown of miR‐212‐5p abolished its suppressive effects on the inflammatory proteins IL‐1*β* and Caspase‐1 associated with the NLRP3 inflammasome (Figure 5i). This effect was observed in both primary macrophages from mice and humans (Figure 5j). To definitively establish a direct interaction between miR‐212‐5p and the 3′‐UTR of *Mettl3*, we conducted luciferase reporter assays in 293T cells. Co‐transfection of the WT luciferase construct of *Mettl3* with miR‐212‐5p mimics resulted in a significant decrease in luciferase activity, while the MUT construct showed no such effect (Figure 5k). This confirmed that *Mettl3* is indeed a direct target gene of miR‐212‐5p. Collectively, these findings suggest that GCs‐EVs effectively reduce the mRNA m^6^A levels of NLRP3 by downregulating Mettl3 expression through the upregulation of miR‐212‐5p.

### 3.7 miR‐212‐5p^KD^ ‐GCs‐EVs failed to mitigate OA progression in the DMM‐induced OA mouse models

**FIGURE 6:**
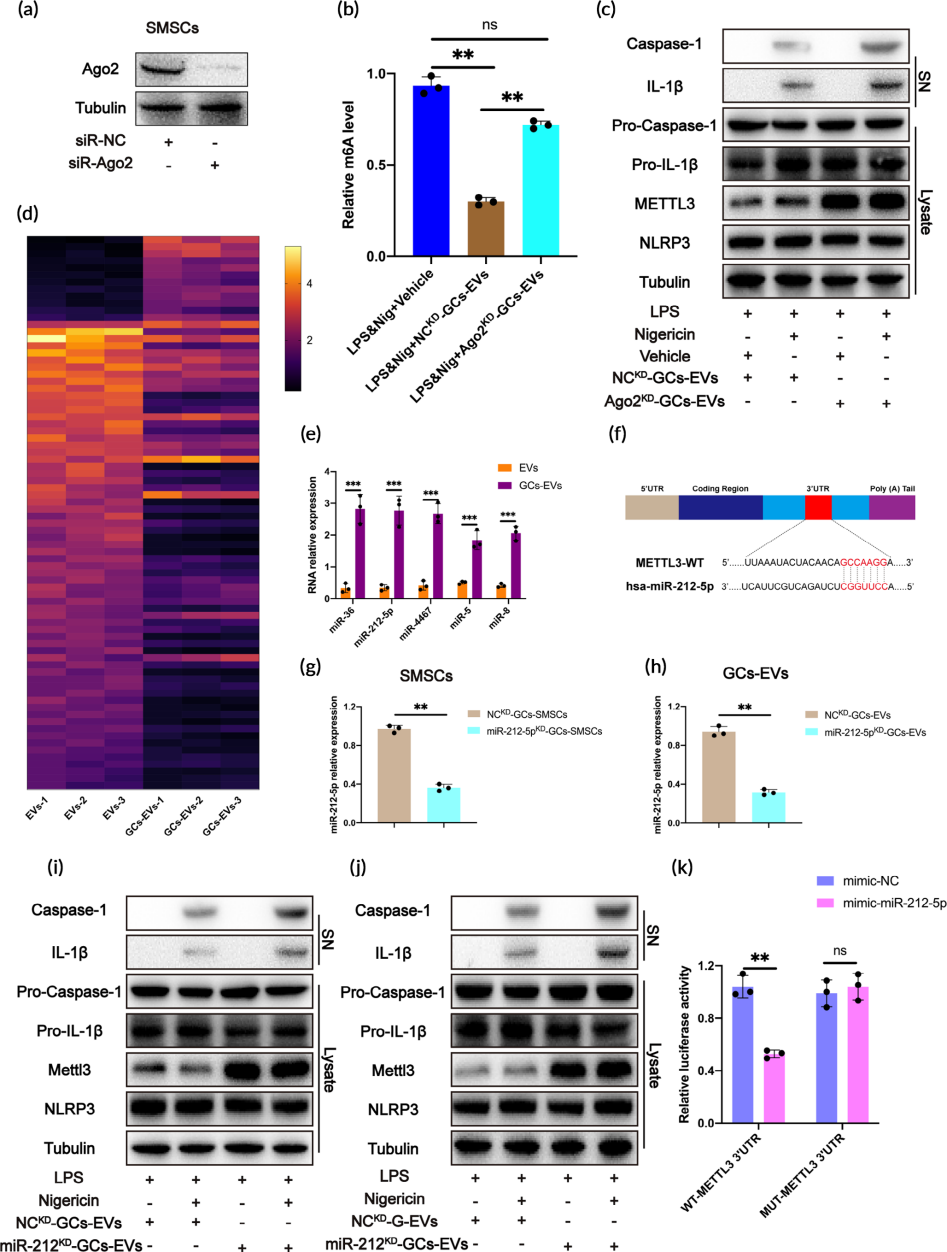
miR‐212‐5p^KD^‐GCs‐EVs failed to mitigate OA progression in the DMM‐induced OA mice models. (a) Severity of DMM‐induced OA mice model as determined by Safranin‐O and Fast Green, and micro‐CT analysis. Expression levels of COL2A1, aggrecan, MMP13 and ADAMTS5 as determined by immunofluorescence staining. Scale bar = 400 μm for Safranin‐O and Fast Green staining; Scale bar = 100 μm for immunofluorescence staining. (b) OARIS scores in each group (*n* = 10, one‐way ANOVA). (c) Osteophyte score of each group (*n* = 10, one‐way ANOVA). (d) Quantification of mean fluorescence intensity of COL2A1, aggrecan, ADAMTS5 and MMP13 in each group (*n* = 3, one‐way ANOVA). (e) Levels of IL‐1*β* and IL‐18 in mice knee tissue as determined by ELISA (*n* = 3, one‐way ANOVA). Data are presented as mean ± SD. ns = no significance, *p < 0.05, **p < 0.01.

After establishing a DMM‐induced OA mouse model to investigate the potential inhibitory effects of miR‐212‐5p in GCs‐EVs on OA progression, knee sections from each experimental group were subjected to Safranin‐O and Fast Green staining. The results demonstrated that the cartilage layer in the GCs‐EVs group was significantly thicker and smoother compared to the miR‐212‐5p^KD^‐GCs‐EVs group (Figure 6a). Moreover, assessment using the OARSI scoring system revealed a significant decrease in OA severity in the GCs‐EVs group compared to the miR‐212‐5p^KD^‐GCs‐EVs group (Figure 6b). Additionally, micro‐CT imaging was employed to evaluate osteophyte formation in mice across all experimental groups. Notably, the osteophytes present in the GCs‐EVs group were substantially smaller than those found in the miR‐212‐5p^KD^‐GCs‐EVs group (Figure 6a). Moreover, the osteophyte score for the GCs‐EVs group was significantly lower than that of the miR‐212‐5p^KD^‐GCs‐EVs group (Figure 6c).

To assess the impact of miR‐212‐5p in GCs‐EVs on ECM proteins, specifically COL2A1 and ACAN, as well as ECM‐degradation‐associated proteases such as MMP13 and ADAMTS5, immunofluorescence analysis was performed on knee sections from mice across various experimental groups. Mice treated with GCs‐EVs exhibited a significant increase in ECM protein levels compared to those receiving miR‐212‐5p^KD^‐GCs‐EVs alone, while the levels of ECM‐degradation‐associated proteases were significantly reduced (Figure 6a, d). These findings suggest that miR‐212‐5p in GCs‐EVs plays a crucial role in inhibiting the expression of ECM‐degrading proteases, thereby protecting ECM proteins from degradation. In addition, the levels of IL‐1*β* and IL‐18 in the knee joint tissues of mice in the miR‐212‐5p^KD^‐GCs‐EVs group were significantly higher than those in the GCs‐EVs group (Figure 6e). Collectively, these findings suggest that the anti‐arthritic efficacy of GCs‐EVs is attenuated upon miR‐212‐5p knockdown.

### 3.8 In the co‐culture system of macrophages and chondrocytes, GCs‐EVs have been shown to enhance the proliferation of chondrocytes

**FIGURE 7:**
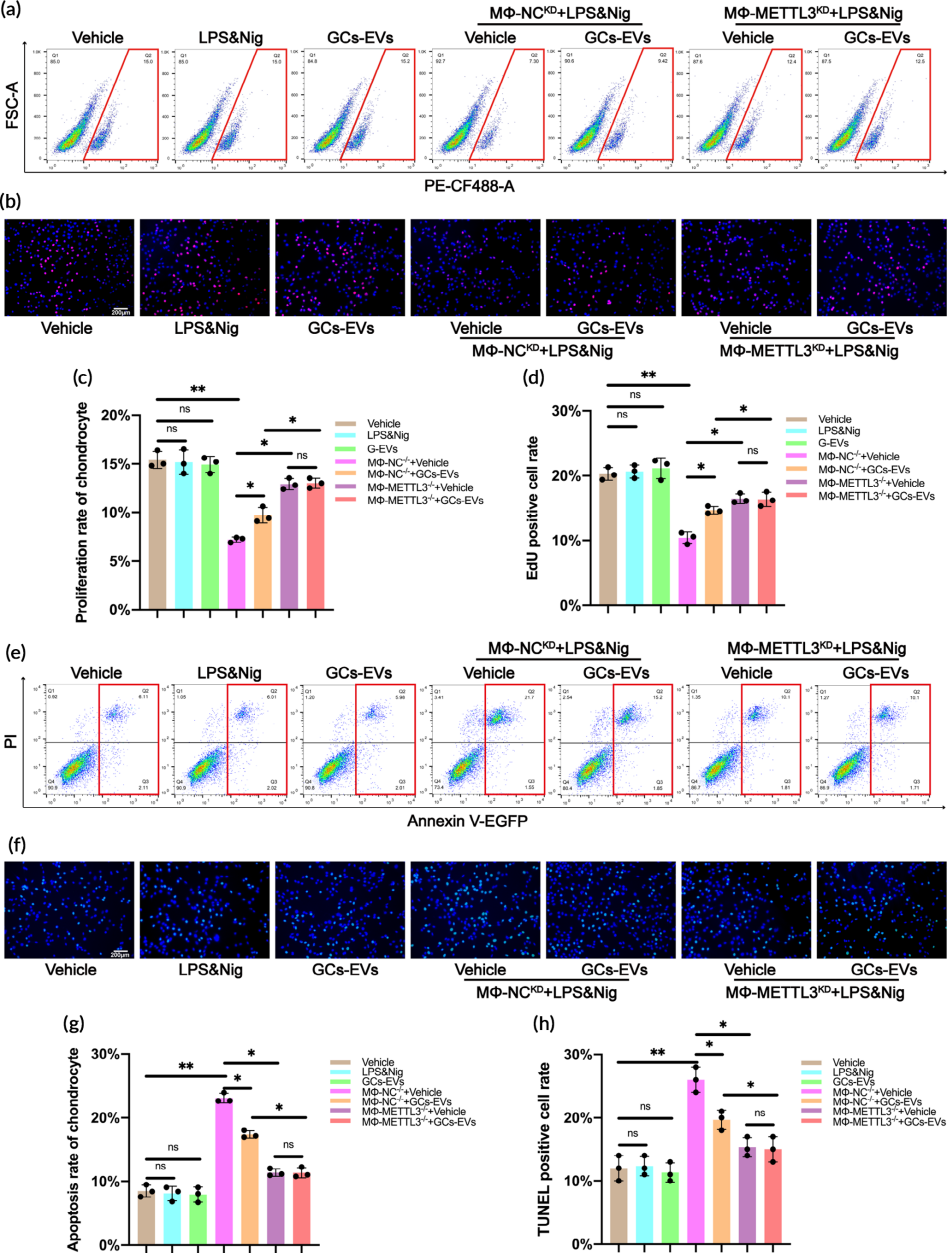
In the system of co‐culture of macrophages and chondrocytes, GCs‐EVs can improve the proliferation rate of chondrocytes and reduce the apoptosis rate of chondrocytes by reducing the level of inflammatory cytokines secreted by macrophages. (a), (c). EdU assay for the proliferation rate of chondrocytes was determined by flow cytometry analysis and the statistical results of flow cytometry analysis for EdU assay (*n* = 3, one‐way ANOVA). (b), (d). The proliferation rate of chondrocytes was observed by immunofluorescence and the EdU positive cell rate was analyzed in each group (*n* = 3, one‐way ANOVA). Scale bar = 200 μm. (e), (g). The Annexin V‐FITC/PI Apoptosis assay for the apoptosis rate of chondrocytes was determined by flow cytometry analysis and the statistical results of flow cytometry analysis for apoptosis analysis (*n* = 3, one‐way ANOVA). (f), (h). The apoptosis rate of chondrocytes was observed by immunofluorescence and the TUNEL positive cell rate was analyzed in each group (*n* = 3, one‐way ANOVA). Scale bar = 200 μm. Data are presented as mean ± SD. ns = no significance, *p < 0.05, **p < 0.01.

To investigate the influence of GCs‐EVs on chondrocyte proliferation mediated by macrophages, we developed a co‐culture system comprising both cell types, as outlined in the methods section. Flow cytometry analysis revealed that treatment with GCs‐EVs alone did not significantly affect chondrocyte proliferation rates (Figure 7a, c). However, in the co‐culture setup, GCs‐EVs notably enhanced the proliferation rates that had been diminished due to macrophage activation (Figure 7a, c). Remarkably, the proliferative effect of GCs‐EVs on chondrocytes was abolished when Mettl3 was knocked down in macrophages (Figure 7a, c). Additionally, an EdU assay conducted on chondrocytes corroborated the flow cytometry findings (Figure 7b, d). The findings indicate that GCs‐EVs may inhibit the NLRP3 inflammasome activity in macrophages, lower the concentration of inflammatory cytokines in the culture medium, and consequently enhance the proliferation rate of chondrocytes.

### 3.9 In the co‐culture system of macrophages and chondrocytes, GCs‐EVs have been shown to decrease the apoptosis rate of chondrocytes

The apoptosis rate of chondrocytes, evaluated through flow cytometry, indicated that treatment with GCs‐EVs alone did not significantly affect chondrocyte apoptosis (Figure 7e, g). However, when macrophages were activated in the co‐culture system, there was a notable increase in chondrocyte apoptosis. GCs‐EVs were effective in mitigating this increase caused by macrophage activation (Figure 7e, g). This protective effect was absent when Mettl3 was knocked down in macrophages (Figure 7e, g). Additionally, TUNEL assays conducted on chondrocytes across all groups corroborated the flow cytometry findings regarding apoptotic chondrocytes (Figure 7f, h). These results suggest that GCs‐EVs may lower chondrocyte apoptosis by diminishing NLRP3 inflammasome activity in macrophages and reducing inflammatory cytokine levels in the medium.

### 3.10 In the co‐culture system of macrophages and chondrocytes, GCs‐EVs enhance the migratory capacity of chondrocytes

**FIGURE 8:**
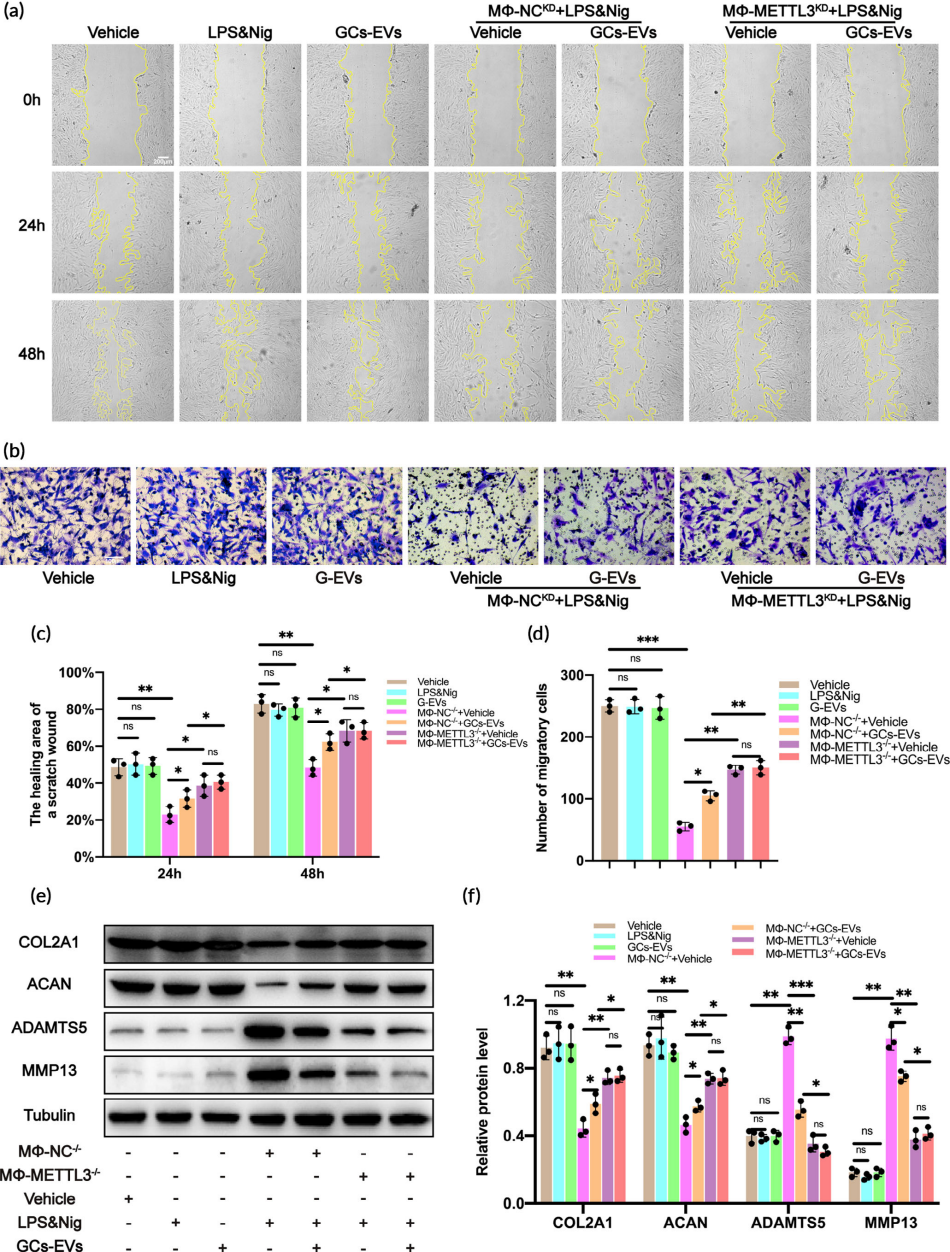
In the co‐culture system of macrophages and chondrocytes, GCs‐EVs can improve the migration ability of chondrocytes and regulate the expression of cartilage matrix proteins by reducing the level of inflammatory cytokines secreted by macrophages. (a), (c). Images of scratch assays on chondrocytes was obtained under a light microscope and the healing area of a scratch wound was analyzed in each group (*n* = 3, one‐way ANOVA). Scale bar = 200 μm. (b), (d). Migration of chondrocytes was observed and quantified by transwell assay and crystal violet was used to stain migrating chondrocytes (*n* = 3, one‐way ANOVA). Scale bar = 100 μm. (e), (f). Western blotting analysis of COL2A1, aggrecan, ADAMTS5 and MMP13 in chondrocytes were detected and quantified in each group (*n* = 3, one‐way ANOVA). Data are presented as mean ± SD. ns = no significance, *p < 0.05, **p < 0.01.

To investigate the impact of GCs‐EVs on the migratory capacity of chondrocytes within co‐culture systems, we performed both cell scratch assays and Transwell assays involving chondrocytes. The cell scratch assays revealed that treatment with GCs‐EVs alone did not significantly alter the migration ability of chondrocytes (Figure 8a, c). Notably, in the activated macrophage co‐culture system, there was a marked reduction in chondrocyte migration; however, GCs‐EVs were able to mitigate this decline caused by macrophage activation (Figure 8a, c). This protective effect was lost when Mettl3 was knocked down in macrophages (Figure 8a, c). The results from the Transwell assays corroborated those from the cell scratch experiments (Figure 8b, d), indicating that GCs‐EVs may improve the migration ability of chondrocytes by reducing the activity of the NLRP3 inflammasome in macrophages and the level of inflammatory cytokines in the culture medium.

### 3.11 In the co‐culture system of macrophages and chondrocytes, GCs‐EVs have the ability to modulate the expression of proteins associated with chondrocytes

Western blot analysis was employed to assess the expression of chondrocyte‐related proteins across different groups. The findings indicated that treatment of chondrocytes with GCs‐EVs alone did not significantly alter the expression of their associated matrix proteins (Figure 8e, f). In contrast, within the co‐culture system, activation of macrophages led to a notable decrease in the expression of chondrocyte ECM proteins, specifically COL2A1 and ACAN, while there was a marked increase in ECM‐degrading proteases, namely MMP13 and ADAMTS5 (Figure 8e, f). As anticipated, the addition of GCs‐EVs to the co‐culture system reversed these regulatory effects (Figure 8e, f). However, when Mettl3 was knocked down in macrophages, the reversing effect of GCs‐EVs on ECM homeostasis was lost. Consequently, there was no significant difference in the regulation of associated chondrocyte matrix proteins between groups treated with GCs‐EVs and those without in the co‐culture systems (Figure 8e, f). We hypothesize that GCs‐EVs could diminish the expression of ECM‐degradation proteases, specifically MMP13 and ADAMTS5, by inhibiting the activity of the macrophage NLRP3 inflammasome and lowering the concentration of inflammatory cytokines in the culture medium. This mechanism may subsequently enhance the expression of chondrocyte ECM proteins, such as COL2A1 and ACAN.

## 4 DISCUSSION

OA is a prevalent degenerative joint disorder primarily affecting the knee. Its pathogenesis involves the breakdown of articular cartilage, formation of osteophytes, subchondral sclerosis, varying degrees of synovial hyperplasia, degeneration of the meniscus, and hypertrophy of the capsular ligaments. These changes can result in pain, deformity, and impaired joint function.^2^, ^59^, ^60^ Research indicates that OA is influenced by inflammation‐related pro‐inflammatory cytokines, particularly IL‐1*β* and IL‐18, which significantly alter the ECM of chondrocytes by upregulating matrix‐degrading proteins.^4^, ^5^ It has been shown that macrophages produce IL‐1*β* and IL‐18 primarily through the activation of the NLRP3 inflammasome.^6^, ^7^ Recent studies have extensively investigated NLRP3 activation, linking it to synovial inflammation in OA.^8^, ^9^ Consequently, targeting NLRP3 to inhibit its activity and subsequently decrease the release of pro‐inflammatory cytokines presents a promising avenue for OA treatment.

MSCs are a type of adult stem cell derived from the mesoderm, found in various tissues such as adipose tissue, bone marrow, dental pulp, skin, and joint synovium.^61^ Among these, SMSCs exhibit exceptional potential for cartilage regeneration due to their tissue‐specific characteristics.^17^ SMSCs are particularly advantageous compared to other MSCs as they can be easily isolated, possess a robust proliferation capacity, and exhibit slower aging processes.^19^ Research indicates that SMSCs maintain their multi‐lineage differentiation capabilities even after multiple passages, specifically beyond the tenth.^19^ Furthermore, recent studies have highlighted the role of extracellular vesicles in facilitating tissue regeneration and addressing various medical conditions. Notably, extracellular vesicles derived from SMSCs have shown significant promise in the treatment of OA.^19^, ^28^, ^62^, ^63^

Preconditioning methods, such as the application of cytokines, pharmaceuticals, hypoxic environments, or physical stimuli, are crucial for enhancing the biological functionality of MSCs and their paracrine activities.^31^ Research has shown that preconditioning SMSCs with lipopolysaccharides significantly enhances the anti‐arthritic properties of their derived extracellular vesicles.^64^ Additionally, MSCs subjected to hypoxic conditions have demonstrated improved reparative effects on spinal cord injuries through their secreted extracellular vesicles.^65^ Furthermore, extracellular vesicles from MSCs pretreated with melatonin have demonstrated superior efficacy in spinal cord injury repair.^66^ While GCs are well‐known for their potent anti‐inflammatory properties and are frequently used to alleviate inflammation in OA patients, their long‐term use is often limited due to associated adverse effects.^32^, ^33^ Therefore, we propose that preconditioning stem cells derived from SMSCs with GCs may amplify the anti‐arthritic properties of their originating extracellular vesicles while mitigating the adverse side effects associated with GCs.

In this research, we present novel evidence demonstrating that GCs‐EVs exhibit superior anti‐arthritic properties compared to EVs. We initially administered both EVs and GCs‐EVs to a mouse model of OA. The results revealed that the cartilage in the GCs‐EVs group was both thicker and smoother, while the volume of osteophyte formation was significantly reduced. Additionally, there was a significant decrease in the levels of IL‐1*β* and IL‐18 in the knee tissue supernatant of the GCs‐EVs group. Given that IL‐1*β* and IL‐18 are downstream inflammatory cytokines associated with the NLRP3 inflammasome, we further investigated the impact of GCs‐EVs in an NLRP3^−/−^ OA mouse model. Our results demonstrated that the aforementioned anti‐arthritic effects were abolished following the knockout of NLRP3. These findings confirm that GCs‐EVs mediate their anti‐arthritic effects through the modulation of NLRP3 inflammasome activity.

Through a series of in vitro experiments, we discovered that GCs‐EVs exert an anti‐arthritic effect by selectively inhibiting the expression of the mRNA methyltransferase Mettl3, which is associated with elevated levels of miR‐212‐5p. This inhibition leads to a reduction in the m^6^A modification of NLRP3 mRNA, consequently diminishing the activity of the NLRP3 inflammasome. To further investigate the impact of GCs‐EVs on human cells, we established a co‐culture system comprising human primary macrophages and chondrocytes. In this system, GCs‐EVs enhanced the proliferation and migration capabilities of chondrocytes while decreasing their apoptosis rate by suppressing the release of inflammatory factors from macrophages. Additionally, GCs‐EVs indirectly elevated the expression of chondrocyte ECM proteins, such as COL2A1 and ACAN, and reduced the levels of ECM‐degrading proteases, including MMP13 and ADAMTS5.

Overall, our study demonstrates that preconditioning with GCs is an effective and minimally invasive technique to enhance the therapeutic potential of extracellular vesicles derived from SMSCs for the treatment of OA. The results suggest that GCs‐EVs may offer a novel, cell‐free strategy to augment the anti‐inflammatory properties of SMSC‐derived extracellular vesicles in OA management. This approach provides a promising and efficient method for addressing OA treatment.

## 5 CONCLUSION

Our research demonstrated that GCs‐EVs significantly mitigated cartilage degeneration and osteophyte development in a mouse model of OA by diminishing the release of inflammatory cytokines from synovial macrophages. This effect is linked to the elevated levels of miR‐212‐5p in GCs‐EVs, which selectively downregulated Mettl3 expression. Consequently, this downregulation resulted in a decreased m^6^A modification of NLRP3 in macrophages, thereby lowering NLRP3 inflammasome activity and enhancing anti‐arthritic effects. Furthermore, in a co‐culture system, GCs‐EVs promoted chondrocyte proliferation and migration while reducing chondrocyte apoptosis by inhibiting the secretion of inflammatory factors from macrophages. This study underscores the superior anti‐arthritic properties of GCs‐EVs over conventional EVs and suggests a promising therapeutic strategy for OA management.

## AUTHOR CONTRIBUTIONS

Z.Y. and S.H. conceived the idea and designed the research project. X.S. and M.Z. fabricated the materials and performed the characterization. X.S. and M.Z. performed the cell studies. X.S. and M.Z. performed the animal studies. X.S., M.Z., Z.Y., and S.H. reviewed and revised the manuscript. All authors approved the submitted vision of the manuscript.

## FUNDING INFORMATION

No benefits in any form have been or will be received from a commercial party related directly or indirectly to the subject of this manuscript.

## CONFLICT OF INTEREST STATEMENT

The authors declare that they have no competing interests.

## Data Availability

The data that support the findings of this study are available from the corresponding author upon reasonable request.
